# Memory Trajectories Before and After First and Recurrent Strokes

**DOI:** 10.1212/WNL.0000000000013171

**Published:** 2022-02-08

**Authors:** Wentian Lu, Marcus Richards, David Werring, Martin Bobak

**Affiliations:** From the Research Department of Epidemiology and Public Health (W.L., M.B.), MRC Unit for Lifelong Health & Ageing (M.R.), and Stroke Research Centre, UCL Queen Square Institute of Neurology (D.W.), University College London, UK.

## Abstract

**Background and Objectives:**

Evidence on timing of memory change after first and recurrent strokes is limited and inconsistent. We investigated memory trajectories before and after first and recurrent strokes in 18 European countries and tested whether the country-level acute stroke care was associated with memory change after stroke.

**Methods:**

Data were from the Survey of Health, Ageing and Retirement in Europe (2004–2019). Incident first and recurrent strokes were identified among baseline stroke-free individuals. Within each country, each participant with incident stroke (case group) was matched with a stroke-free individual (control group) using propensity score matching. We applied multilevel segmented linear regression to quantify acute and accelerated memory changes (measured by the sum score of immediate and delayed word recall tests; 0–20 words) before and after first and recurrent strokes in both groups. Associations between stroke and memory were compared between countries with different levels of acute stroke care indicators.

**Results:**

The final analytical sample included 35,164 participants who were stroke-free at baseline (≥50 years). A total of 2,362 incident first and 341 recurrent strokes between 2004 and 2019 were identified. In case groups, mean acute decreases in memory scores were 0.48 (95% confidence interval [CI] 0.31, 0.65) and 1.14 (95% CI 0.80, 1.48) words after first and recurrent stroke, respectively, independent of a range of confounders. No such acute decreases were observed in the control group after a hypothetical nonstroke onset date. In both groups, memory declined over time but decline rates were similar (−0.07 [95% CI −0.10, −0.05] vs −0.06 [95% CI −0.08, −0.05] words per year). The mean acute decreases in memory scores after first and recurrent strokes were smaller in countries with better access to endovascular treatment.

**Discussion:**

We found acute decreases but not accelerated declines in memory after first and recurrent strokes. Improved endovascular therapy might be associated with smaller memory loss after stroke but more evidence based on individual-level data is needed. More effort should be made in early assessment and intensive prevention of stroke among the ageing population and promoting access to and delivery of acute stroke care among patients with stroke.

Cohort studies worldwide have suggested that following stroke there is cognitive decline affecting verbal memory, verbal fluency, temporal orientation, processing speed, executive function, and global cognition over time.^1-20^ However, the exact timing of the association remains unclear. Several cohort studies have estimated cognitive transitions before and after first stroke; the results were mixed, perhaps due to disparities in cognitive measures. One study (2010–2012) in the United States found that cognition declined nearly twice as fast after a first stroke than before.^4^ One study (2002–2015) in England suggested both acute decreases and accelerated declines in global cognition, verbal memory, verbal fluency, and temporal orientation after first stroke.^9^ Another US study (1998–2008) indicated an acute decrease in memory after first stroke, but at a rate of decline similar to that observed prestroke.^3^ One study between 2003 and 2013 in the United States reported acute decreases in global cognition and verbal memory after first stroke, but no difference between pre- and poststroke rate of decline in verbal memory.^2^

Studies also found that patients with stroke recurrence had greater decreases in multiple domains of cognition than those without stroke recurrence.^12,14,15^ However, these studies had limited years of follow-up (≤3 years) and relatively few repeated measures of cognition (≤3 times).

Data from the Survey of Health, Ageing and Retirement in Europe (SHARE) provide an opportunity to investigate associations between incident strokes and cognition in multiple countries.^21^ As there is evidence of inequalities in access to, and delivery of, acute stroke care across European countries,^22^ we also investigated whether improved implementation of acute stroke care (stroke unit [SU] care, IV thrombolysis [IVT], and endovascular treatment [EVT]) is associated with less cognitive decline after stroke onset. We estimated memory trajectories before and after first and recurrent strokes, tested the association between stroke and memory change using matched stroke-free individuals, and whether this association was independent of a wide range of potential confounders; and tested whether cross-country differences in acute stroke care were associated with memory change after stroke.

## Methods

### Sample

We used data from 18 countries with at least 3 waves of data collection and common variables for repeated measures of cognition and stroke, as well as other baseline covariates in SHARE.^21^ SHARE is a panel database of micro data on health, socioeconomic status, and social and family networks among older adults aged 50 years and over in most countries of the European Union and Israel. Participants were selected randomly from sampling frames chosen as the best available frame resources in each country (mostly population registers). We divided these countries into 2 groups based on their ranks in providing acute stroke care including SU, IVT and EVT (see below).^22^

We included SHARE participants (waves 1, 2, and 4–7, 2004–2019) who were stroke-free at baseline. The retrospective survey (wave 3) focusing on participants' life histories was not included in our study. We also excluded participants aged <50 years at baseline (core participants' younger/new partners), those who joined in baseline wave only, baseline booster sample, and those who self-reported diagnosed dementia or Alzheimer disease at baseline. Each participant with incident first stroke (case group) in each country was matched with a population-based stroke-free individual (control group; matching ratio: 1:1) using propensity score matching. Participants in the control group were at a high risk of having stroke (see below). See eFigure 1, links.lww.com/WNL/B693, for the procedure of sample selection.

### Standard Protocol Approvals, Registrations, and Patient Consents

SHARE was approved by the Ethics Committee of the University of Mannheim and the Ethics Council of the Max Planck Society.^21^ All participants provided written informed consent.^21^

### Memory

Episodic verbal memory was assessed in a standardized manner via 2 conditions of a word-recall test: participants listened to series of 10 words and then were asked to immediately recall as many words as possible in any order and allowed up to 1 minute for recall (immediate recall: range 0–10 words); after a few minutes, participants were asked to recall as many of the original words as possible in any order (delayed recall: range 0–10 words); in between, participants were asked to take several other cognitive tests and were not informed of the delayed recall test ahead. We then summed the 2 scores (range 0–20). Time-varying (at each wave) scores were used to permit modeling of change in memory over the follow-up period.

We did not consider other measures of cognition such as the date-naming test for general orientation, animal-naming test for verbal fluency, arithmetical calculations for numeracy, and the serial 7s test for working memory, due to disparities in these measures across waves and countries (see Discussion).

### Participants With Incident Stroke

Self-reported questions whether participants have had clinically diagnosed stroke (no information on stroke types; yes/no) at baseline wave, and whether they have had clinically diagnosed stroke (yes/no) and the number of strokes occurred since last interview (1/2/≥3/unknown) in waves 2 and 4–7, were used to identify incident first and recurrent strokes. Ages and dates of an interview in each wave of data collection were used to calculate the ages of the first and the second stroke onsets (rounding to one decimal place). In this study, we only considered stroke recurrence in terms of onset of a second stroke. The first and the second stroke onsets could be in the same or different waves of data collection. Thus, we made different types of calculation for the ages of the first and the second stroke onsets in different scenarios (eFigure 2, links.lww.com/WNL/B693).

### Follow-up Years

We calculated years of follow-up for each participant using the age in each wave of data collection minus the age at first stroke onset. The time of the first stroke onset was set as zero. Negative and positive values designated prestroke (−16.0 to 0.0) and poststroke (0.0–16.0) years. The gap between first and recurrent stroke ranged from 0.3 to 5.5 years.

### Stroke Unit, IV Thrombolysis, and Endovascular Treatment

Countries were classified using the number of SUs per 1,000 annual incident ischemic strokes (cutoff ≥1.6 units), the annual proportion of patients with incident ischemic stroke treated with IVT (cutoff ≥10.4%), and the annual proportion of patients with incident ischemic stroke treated with EVT (cutoff ≥3.2%), based on a survey of acute stroke care in 44 European countries in 2015/2016.^22^ Countries ranked above these cutoffs were classified as having a moderate to high level of quality of acute stroke care.^22^ We did not use the target number/rate of SU/IVT/EVT that this survey provided as cutoffs (i.e., SU ≥3 units, IVT ≥18%, and EVT ≥5%) as only 2 or 3 European countries had achieved these targets.^22^ Table 1 shows countries' ranks by SU, IVT, and EVT.

**Table 1:**
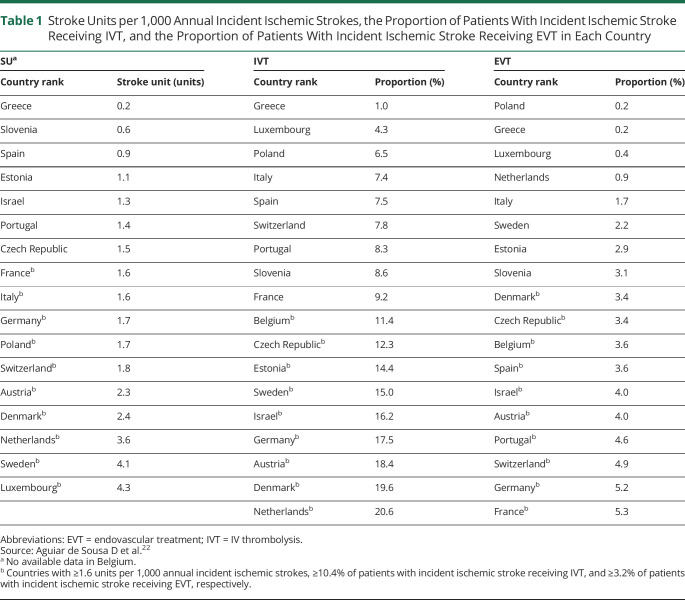
Stroke Units per 1,000 Annual Incident Ischemic Strokes, the Proportion of Patients With Incident Ischemic Stroke Receiving IVT, and the Proportion of Patients With Incident Ischemic Stroke Receiving EVT in Each Country

### Covariates

Based on previous studies,^2,3,9,23^ baseline variables for sex (men/women), year of birth, marital status (married/cohabiting, single/divorced/separated/widowed), education (highest educational degree obtained: tertiary education or above, secondary education, primary education, less than primary education), wealth (total family assets: quintiles), smoking (non-/ex-/current smokers), frequency of alcohol consumption during the last 6 months (never/rare/often), vigorous exercise during the last 2 weeks (yes/no), high-risk depressive symptoms (EURO-D ≥4^24^), obesity (body mass index ≥30.0^25^), contact with friends and relatives at least once per month (yes/no), self-reported diagnosed high blood pressure, heart attack or any other heart problems, diabetes and hearing impairment (diagnosed by doctors: yes/no), height (cm), and the respondent status of mortality during follow-up (alive/died/unsure) were selected as potential confounders.

### Statistical Methods

All SHARE countries have complex survey designs. Baseline weights were used to adjust for bias due to sampling design when conducting all analyses.

Multiple imputation based on chained equations was employed in each country to handle missing data for memory and confounders.^26^ The percentages of missingness in baseline memory and confounders ranged from 0.1% to 16.8% (eTable 1, links.lww.com/WNL/B693). All covariates, repeated measures of memory and stroke diagnosis, and baseline weights were included in each imputation model. We used the imputed values of memory and covariates but only the original values of stroke diagnosis for the analyses. Fifty imputed datasets were generated in each country. We combined imputed datasets for all countries to run analyses.

We conducted univariate analysis to compare the baseline sample characteristics between participants with incident stroke and stroke-free individuals. Linear, logistic, ordered logistic, and multinomial logistic regression were employed.

For the main analysis, we first applied multilevel segmented linear regression^27^ to assess the longitudinal relationship between follow-up years (“time”; pre- and poststroke years for first stroke and stroke recurrence) and memory in participants with incident stroke in all countries, allowing for random intercepts and slopes. For each individual, we generated a time-varying variable “post,” coding 0 for prestroke years (reference), 1 for poststroke years after first stroke, and 2 for poststroke years after stroke recurrence. Our model included time, post, and interaction between time and post. We also fit country- and individual-level clustering to the regression model. The coefficient for time estimates the secular trend in memory during follow-up, independently of stroke onset; the coefficient for post estimates the acute change in level in memory trajectory after first stroke and after stroke recurrence; and the interaction between time and post reflects the change in slope in memory trajectory after first stroke and after stroke recurrence (i.e., accelerated linear rate of decline in memory). See an example of the linear equation to be analyzed without considering confounding adjustment (eMethods, links.lww.com/WNL/B693).

We built fully adjusted models controlling for all covariates. The Wald test^28^ was applied to check the significance of the time–post interaction (at the 95% level) and help build the final models. We did not consider the nonlinear effect of time, as the Wald test suggested no improvement in model fit when adding quadratic time in regression model (*p* values >0.05).

Second, to test the independent association between stroke onset and memory over time, we used propensity score matching to match each participant with incident stroke (case group) with a population-based stroke-free individual (at a high risk of having stroke; control group) in each country, based on similar propensity scores obtained from a logistic regression model adjusting for all confounders.^29^ The nearest neighbor matching was used (caliper bound = 0.04, mean bias = 1.6%–3.8%). Bootstrap estimation was performed with 1,000 replications. As a result, the observed baseline sample characteristics became very similar between 2 groups. To compare memory trajectories before and after a hypothetical nonstroke onset date in stroke-free individuals, each individual was allocated nonexposure data, which were the ages at first stroke and stroke recurrence of his or her matched treated participant. We assessed the unadjusted relationships between follow-up years (pre- and poststroke years) and memory using multilevel segmented linear regression in both groups. We also estimated memory trajectories before and after first and recurrent strokes in both groups, assuming the recurrent stroke occurred at 2.5 years after first stroke. The 2.5 years was the mean gap (kurtosis = 2.4; skewness = 0.3) between first and recurrent stroke in all countries.

Finally, we reran the multilevel segmented linear regression in participants with incident stroke in all countries, considering the interaction between “post” and “moderate or high SU/IVT/EVT category (group).” This is to test whether the country-level acute stroke care category modified memory change after stroke. The Wald test was also applied to check the significance of the post–group interaction (at the 95% level) and help build the final models. If the post–group interaction was statistically significant and the Wald test suggested a better fit when adding this interaction in the regression model, we estimated the memory trajectories before and after first and recurrent strokes in each acute stroke care category to aid the interpretation of the post–group interaction, assuming the recurrent stroke occurred at 2.5 years after first stroke.

### Sensitivity Analyses

To check clinical significance, we estimated having first/recurrent stroke is equivalent to becoming cognitively how many years older, using nonlinear combinations of coefficients for secular trend and changes in level after first and recurrent strokes. We also ranked countries by Active Ageing Index (AAI).^30^ AAI reflects older adults' psychosocial and physical capacities and countries' economies and welfare states, which might be a strong confounder for the association between EVT and poststroke memory change. We repeated main analyses, considering the interaction between post and EVT and controlling for countries' ranks by AAI. In addition, we regenerated beta coefficients by standardizing memory scores relative to controls at each wave in each country, and repeated main analyses. Using standardized outcome measures can help compare research findings across studies and allow clinicians to build up evidence for treatments.

All analyses were performed using Stata MP 16,^31^ with a *p* value threshold of <0.05 for statistical significance.

### Data Availability

SHARE data are freely available to researchers.^21^ Data of our study can be obtained on request.

## Results

Table 2 presents the baseline sample characteristics in participants with incident stroke and stroke-free individuals. The final analytical sample was 35,164, consisting of 2,362 participants with incident first stroke and 32,802 stroke-free individuals. Among those with incident first stroke, 341 (14.44%) participants with recurrent stroke were identified. Compared with stroke-free individuals, participants with incident stroke tended to be older, men, unmarried, less educated and physically inactive, deceased during follow-up, and have lower mean memory scores, lower levels of wealth, high-risk depressive symptoms, high blood pressure, heart attack or any other heart problems, obesity, diabetes, and hearing impairment. However, there were larger proportions of nonsmokers and nonalcohol consumers among participants with incident stroke than stroke-free individuals. Participants with incident stroke were more likely to come from countries with a moderate to high level of IVT and EVT care.

**Table 2:**
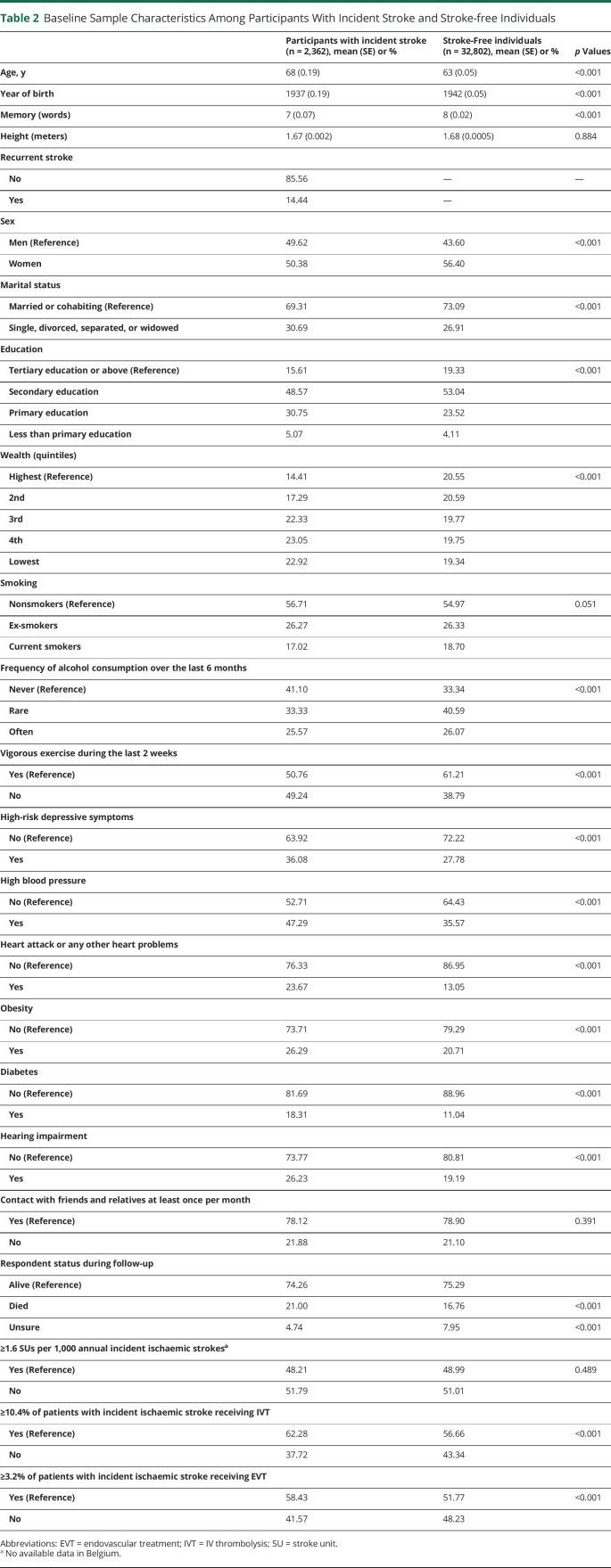
Baseline Sample Characteristics Among Participants With Incident Stroke and Stroke-free Individuals

Table 3 presents memory trajectories before and after first and recurrent strokes in participants with incident stroke. We built fully adjusted models with (model A) and without (model B) the time–post interaction (i.e., change in slope). In model A, there were significantly negative relationships between follow-up years and memory, suggesting that memory declined over time (whether pre- or poststroke onset). There were significant acute decreases in memory (i.e., changes in level) after both first and recurrent strokes. Compared with memory before stroke, we found mean acute decreases in memory score of 0.50 (95% confidence interval [CI] 0.33, 0.68) and 1.06 (95% CI 0.72, 1.40) words after first and recurrent stroke, respectively. The acute memory decreases remained significant after adding the time–post interaction (i.e., change in slope) to the model. However, the time–post interaction was nonsignificant (model B: *p* values = 0.855 and 0.088), suggesting that memory decline rates were similar before and after stroke. The Wald test showed no improvement in model fit after adding this interaction (*p* value = 0.211), suggesting that model A was a better fit.

**Table 3:**
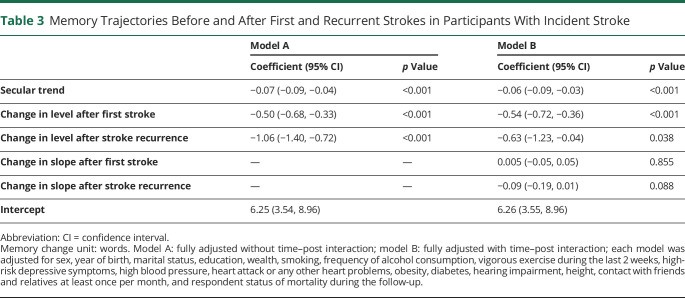
Memory Trajectories Before and After First and Recurrent Strokes in Participants With Incident Stroke

**Figure 1:**
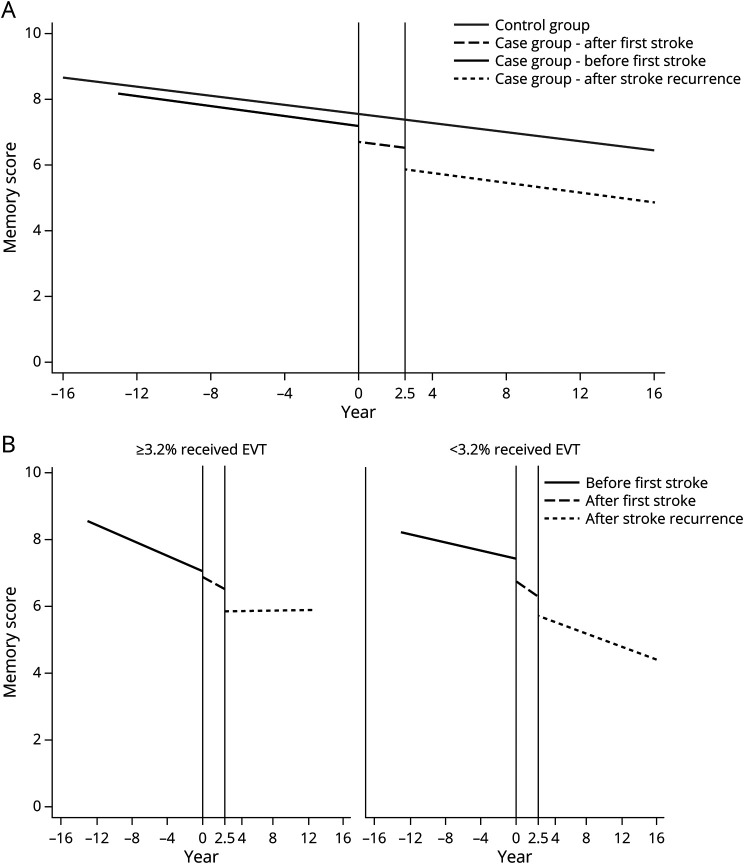
Memory Trajectories Before and After First and Recurrent Strokes Memory trajectories before and after first and recurrent strokes in participants with incident stroke and 2 endovascular treatment groups (B). Memory change unit: words.

Table 4 and Figure 1A present memory trajectories before and after first and recurrent strokes in case and control groups. In both groups, memory declined over time (decreasing by 0.07 [95% CI 0.05, 0.10] vs 0.06 [95% CI 0.05, 0.08] words per year). Mean memory trajectory was worse in the case group than the control group but the acute decreases between pre- and poststroke memory were only significant in the case group (decreasing by 0.48 [95% CI 0.31, 0.65] words after first stroke and by 1.14 [95% CI 0.80, 1.48] words after recurrent stroke), suggesting an independent acute effect of stroke on memory decrease.

**Table 4:**
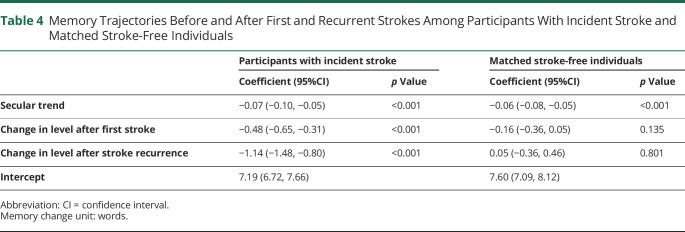
Memory Trajectories Before and After First and Recurrent Strokes Among Participants With Incident Stroke and Matched Stroke-Free Individuals

Table 5 shows memory trajectories before and after first and recurrent strokes, considering the interaction between poststroke level decrease and indicators of acute stroke care availability. Only the interaction with EVT category was statistically significant, and the Wald test suggested a better model fit after adding this interaction (*p* = 0.0005). This interaction suggested greater acute decreases in memory score after first and recurrent strokes in countries with a low level of EVT care. Figure 1B illustrates this finding by showing memory trajectories in the 2 EVT groups. The intercept gap between prestroke trajectory and the trajectory after first and recurrent stroke was larger in countries with a low level of EVT care than those with a moderate to high level of EVT care. After stroke onset, the memory trajectories also declined faster in countries with a low level of EVT care.

**Table 5:**
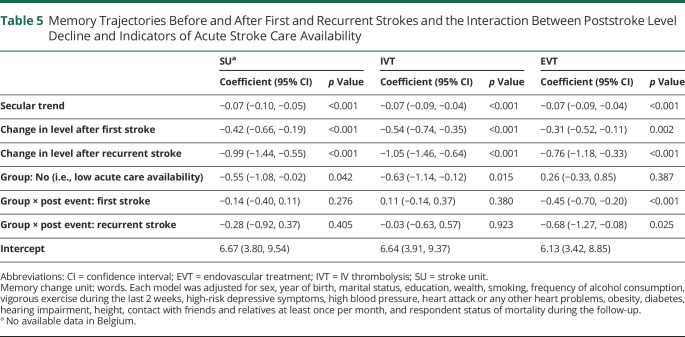
Memory Trajectories Before and After First and Recurrent Strokes and the Interaction Between Poststroke Level Decline and Indicators of Acute Stroke Care Availability

For sensitivity analyses, in participants with incident stroke, having first and recurrent stroke is equivalent to becoming cognitively 6.4 (95% CI 2.6, 10.3) and 15.3 (95% CI 7.5, 23.2) years older, respectively. There were no such changes in the control group (eTable 2, links.lww.com/WNL/B693). AAI was a confounder: middle- and bottom-ranked countries had worse mean memory trajectories than top-ranked countries. The interaction with EVT category was negative and significant (eTable 3, links.lww.com/WNL/B693). In the case group, the mean acute decrease in memory was 0.13 (95% CI 0.08, 0.18) and 0.31 (95% CI 0.22, 0.41) SDs after first and recurrent stroke, respectively. In the control group, the changes in memory after first and recurrent stroke dates were nonsignificant (eTable 4, links.lww.com/WNL/B693).

## Discussion

In this large multicountry dataset, we found acute decreases in memory after incident first and recurrent strokes in comparison to matched stroke-free individuals, whereas memory decline rates were similar. These associations were independent of a wide range of potential confounders. Acute memory decreases were smaller in countries with moderate to high levels of EVT care, suggesting that improved EVT might be associated with smaller memory loss after stroke.

Our finding of the independent association between stroke and acute decrease in memory suggests a central causal role of the stroke event itself, as opposed to the underlying vascular risk factors. Patients may have pronounced processing speed deficits soon after stroke.^32^ Decreased processing speed appears to underlie poststroke dysfunction in episodic memory.^33^ However, we did not find an accelerated memory decline after first and recurrent strokes. Stroke can contribute to cognitive impairment in the acute phase, but having a long-term cognitive impairment may require additional cerebral pathology.^34^ For example, the long-term decline in episodic memory may be caused by β-amyloid–induced hippocampus atrophy.^35^

Our finding of an acute decrease but not accelerated decline in memory after first stroke is consistent with those in the United States,^2,3^ although the memory measures were different in these studies. One of the US studies generated a composite memory score combining immediate and delayed recall tests, self-rated memory, and the Informant Questionnaire on Cognitive Decline.^3^ The other US study used a delayed recall test for evaluating verbal memory.^2^ The memory measure employed in the English study was the same as that in the current study.^9^ However, the English study suggested both an acute decrease and accelerated decline in memory after first stroke.^9^ The inconsistent findings might be due to differences in the study population, the timing of follow-up, and the method of identification of participants with incident stroke.

Studies also reported greater memory decreases within 1,^15^ 2,^12^ and 3^14^ years after stroke recurrence. Our study suggested that these memory changes after stroke recurrence might be acute and that there is a dose–response effect of stroke on memory decrease. Our findings highlight the importance of multiple strokes in the pathogenesis of dementia.^12,36^ A meta-analysis based on 73 studies worldwide indicated that there would be 10% of patients developing new dementia soon after a first stroke and more than 33% of patients would have dementia after stroke recurrence.^37^

The acute decreases in memory scores in our study are likely to be clinically meaningful. A decrease of 0.5–1.5 words in memory score after first and recurrent strokes (Table 4) should not be treated as being trivial. Such decreases can have a major effect on cognition as patients might become cognitively 6 and 15 years older immediately after first and recurrent stroke, respectively (eTable 2, links.lww.com/WNL/B693). This might explain why 10% of patients can have a high risk of developing new dementia soon after first stroke.^37^

Our findings suggest that better implementation of EVT in acute stroke care was associated with the poststroke memory change. However, among European countries included in our study, only France and Germany reached the recommended target rate in 2016 (≥5% of patients with ischemic stroke received EVT); by contrast, countries such as Poland and Greece achieved this proportion of EVT in only 0.2% of patients with ischemic stroke (Table 1). The main reasons for not providing EVT were lack of specifically trained personnel, facilities, and costs.^22^ Countries with low levels of SU and IVT had worse memory trajectories than those with moderate to high levels of SU and IVT. However, the country levels of SU and IVT were not associated with poststroke memory changes (Table 5). SU classification is heterogeneous across European countries. Estimations of SU were based on an assumption of equal distributions of SU within countries.^22^ Evidence using other indicators of SU care, such as the number of SU beds or patients treated in one SU, is needed.

This study has several strengths. It included recent data from 18 European countries and examined time trends over a period of up to 16 years before and after first stroke. We used closely matched stroke-free individuals drawn from the same populations to test whether stroke was an independent risk factor for memory. We applied advanced statistics including multilevel modeling and segmented regression to predict memory trajectories before and after first and recurrent strokes, as well as comparing memory changes in poststroke years in countries with different levels of acute stroke care.

Our study has several limitations. First, there might be residual confounding effects. Other risk factors such as *APOE* genotype, atrial fibrillation, or unmeasured aspects of care were not considered due to unavailability of the data. However, our models controlled for many potentially modifiable risk factors for dementia, which are associated with an estimated 35% of the population attributable fraction of dementia worldwide.^23^ The association between EVT and poststroke memory change could also be confounded by other country-level factors, such as countries' healthy aging status. However, sensitivity analyses suggested that the interaction with EVT category was still negative and significant after controlling for AAI (eTable 3, links.lww.com/WNL/B693).

Second, we were unable to include other domains of cognition such as verbal fluency or temporal orientation in these selected SHARE countries, due to the disparities in cognitive measures across waves and countries. For example, SHARE started the Serial 7s test only from wave 4, and countries such as Luxemburg and Portugal had less than 3 waves of data collection on numeracy, general orientation, and verbal fluency, which were not suitable for our research question. Future studies could select fewer SHARE countries and focus on other domains of cognition for the assessment of cognitive transitions before and after stroke onset. The SHARE study did not use any formal norms in their memory tests. A recent Delphi expert study suggested that verbal memory and processing speed are much less challenging to be captured in a cross-cultural cognitive test than language, social cognition, executive functioning, visuospatial functioning, working memory, and orientation.^38^ Although SHARE used different languages for cognitive tests, each interviewer's primary language was considered during interview. Translations were made based on a language switching protocol, a language management system, training for neuropsychologists, translators, and interviewers, and collaborations between countries.^39^

Third, we relied on self-reported clinically diagnosed stroke. The calculations for the ages at stroke onsets might not be accurate due to incorrect reports and missing values in the number of strokes. Some recall bias is likely from impaired memory itself and could lead to misclassification of first incident and recurrent strokes and their timing. However, our findings were consistent with those based on both self-reported and medical records of clinically diagnosed stroke in the United States.^2,3^ A US study found good agreement between self-reported questionnaires and medical record data for stroke.^40^ In general, questionnaire data are valuable for the study of acute-onset diseases (e.g., myocardial infarction or stroke) and chronic conditions requiring ongoing management (e.g., diabetes or hypertension).^43^ We identified approximately 7% of participants with incident stroke during an up to 16-year follow-up. For comparison, studies based on self-reported stroke identified around 9% (US)^3^ and 5% (England)^9^ of participants with incident stroke during up to 10-year and 12-year follow-up, respectively. The stroke recurrence rate at 5.5 years after first stroke among stroke survivors was around 19.5% in our study. In a recent systematic review, the pooled recurrence rate of 32 studies in stroke survivors (>50 years) was 18.1% at 5 years after first stroke.^41^ The incidence rate of stroke recurrence in our data is consistent with external data. Furthermore, SHARE data have been treated as a valid data source to estimate the economic burden of stroke across European countries.^42^ Although self-reported stroke diagnosis is not a perfect measure, we are confident that it is adequately valid for the purpose of this analysis. If anything, a recall bias leading to underreporting of strokes due to impaired memory would underestimate the strength of the main association rather than lead to spurious findings.

Fourth, we only imputed item nonresponse instead of nonresponders in each wave. During follow-up, 21% of participants died and 4.74% participants' status was “unsure” (Table 2). Those participants tended to have more severe stroke than those who remained in the sample. In main analyses, we controlled for participants' respondent status during follow-up, and also applied multilevel modeling—an approach to handling attrition, wave nonresponse, and unequal time spacing.^43^ Although statistical strategies can to some extent address the potential bias caused by missingness, they are not perfect and our findings might underestimate the association between stroke onset and memory changes.

Finally, country ranks by SU, IVT, and EVT were preliminary, given the inconsistent data quality across countries, as well as the lack of a uniform definition of SU and a well-established indicator for population density in each country when calculating the estimates of SU, IVT, and EVT in the original studies.^22^ The SHARE data contain no information on stroke types, while the SU, IVT, and EVT apply to ischemic strokes.^22^ Because a majority (around 85%) of strokes are ischemic,^44^ the association between markers of acute stroke care at country level (i.e., SU, IVT, and EVT) and poststroke memory change and the modifying effect of EVT on poststroke memory might have been underestimated. This limitation of the data, however, does not contradict the general need for providing high quality of acute stroke care to as many patients as possible.^45^

The findings of our study highlight the need to facilitate the European Stroke Action Plan for 2018–2030.^46^ National plans for stroke encompassing the entire chain of care from primary prevention to health outcomes after stroke is necessary. More effort should be made in early assessment and intensive prevention of stroke among the general aging population, as well as promoting access to and delivery of SU care along with IVT and EVT among patients with stroke. These strategies may be important for lessening memory decline and ultimately dementia burden among high-risk older adults and stroke survivors.

We found acute decreases but not accelerated declines in memory after first and recurrent strokes. Our study confirms that stroke has an independent acute effect on memory and suggests a causal role of stroke in the progression of cognitive decline. The country-level data, although not entirely complete, suggest that improved endovascular therapy might be associated with smaller memory loss after stroke but more evidence based on individual-level data is needed.

## Glossary

AAI: Active Ageing Index
CI: confidence interval
EVT: endovascular treatment
IVT: IV thrombolysis
SHARE: Survey of Health, Ageing and Retirement in Europe
SU: stroke unit
